# The characteristics and influencing factors of spatial network of city-based innovation correlation in China: from the perspective of high tech zones

**DOI:** 10.1038/s41598-023-43402-5

**Published:** 2023-09-28

**Authors:** Hong Zhang, Lili Jiang, Jia Zhou, Nanchen Chu, Fengjiao Li

**Affiliations:** 1 College of Geography Science, Harbin Normal University, Harbin, 150025 China; 2 University of Chinese Academy of Sciences, Beijing, 100049 China

**Keywords:** Socioeconomic scenarios, Sustainability

## Abstract

In the context of “space of flows”, city-based innovation correlation in driving economic growth is no longer limited to the traditional hierarchical structure. It is of great significance to explore Chinese cities innovation association network from the perspective of high-tech zones which gather a large number of innovation resources. Here our report is to provide new ideas for improving the innovation capability of high-tech zones and accelerating the construction of Chinese high-quality innovation system. Here we take 142 cities with high-tech zones as research samples, and explore the characteristics and influencing factors of spatial network of city-based innovation correlation in China, through modified gravity modelsocial, network analysis and QAP analysis. The results show that city-based innovation network is not closely connected, the number of redundant connection channels is low efficiency, showing a four-level spatial pattern of “Z” shaped spindle. Among them, degree centrality of cities in eastern China is higher than that in the western region, the core cities in central China play a bridging role, and western remote cities are easily affected by related cities. Moreover, there are four innovation cohesion subgroups, including the northern hinterland subgroup, the eastern coastal subgroup, the southern subgroup and the western cooperation subgroup. Furthermore, the results of the influencing factors analysis show the differences in administrative level, economic development level, openness to the outside world, and investment in technology are conducive to the innovation association between cities, while the similarities in spatial adjacency and industrial structure will promote the strong innovation association between cities.

## Introduction

The rapid development of information technology has broken the traditional interaction mode between cities. The flow of elements across time and space produces high-frequency and diversified urban networks, and creates space of flows that shocks the logic of original space of places. This transformation also promotes the theoretical basis of urban interaction from “central place theory” to “flow space theory^1^.” The elements of flow include information flow, innovation flows, capital flow, etc.. In the context of “space of flows”, these flows primarily exist in a connected manner, and innovation flows based on connections become the fundamental units of innovative networks, gradually giving rise to a spatial pattern of innovation networking^2^.Freeman first proposed the concept of innovation networks, suggesting that innovation networks are internal innovative processes based on institutional arrangements within a system^3^. Innovation networks can be regarded as the flow and interaction among various elements, forming nodes and the connections between nodes. These node elements include universities, enterprises, research institutions, and other sectors, with innovation as their main purpose. The innovation network is form by the intertwining of a three-helix structure consisting of scientific research institutes (science and technology chain), high-tech enterprises (industry chain) and regional governments (administrative chain), which is coupled by National High Tech Industrial Development^4,5^. National High Tech Industrial Development Zones (HTZs) is the spatial carrier of high-tech enterprises and industries^5^, and is a cutting-edge technology and intelligence-intensive area for key development under the support of national policies in China^6^. According to the statistics from the Chinese Ministry of Science and Technology Torch high-tech industry development centers, as of 2020, there are 169 HTZs in China, with a gross domestic product (GDP) of 13.56 million RMB, accounting for 13.3 percent of the national GDP (10.16 million RMB). Simultaneously, as the interactive lotus root complex of innovation cluster and industrial cluster, HTZ is a kind of actor cyberspace that integrates regional urban space space and national global flow space^7^.

The construction methods of innovation correlation network mainly divide into two categories: direct construction and indirect analog. The first is to directly build urban innovation correlation network based on factor flow. Specifically, it can be divided into actual flow elements and virtual flow elements. In terms of the actual flow factors, the flow data of innovative talents such as entrepreneurs^8^, and scientists^9,10^ are mainly used to build the urban innovation correlation network, and the inter-city technology exchange and knowledge transmission promoted by the flow of talents are explored. In terms of virtual flow elements, which are concerned, researches are mainly carried out through knowledge flow data such as scientific and technological achievements. The patent database of the State Intellectual Property Office of P. R. China (SIPO) and scientific and technological literature databases such as CNKI and WOS are often used as data sources to mine joint patent data^11^, patent right transfer data^12^ and co-authored data^13^ to directly build a knowledge innovation association network. The second is to build an innovative correlation network indirectly through the relationship model. scholars try to transform the multi-dimensional innovation attribute data into the association data by constructing a relationship model, so as to indirectly simulate the urban innovation association network, among which the chain model and the gravity model are more common. As far as the chain model is concerned, it refers to the construction of inter-city correlation network through the relationship between internal organization and external cooperation. For example, The organizational connections such as the branch relationship of the headquarters of innovative enterprises or high-tech enterprises^14^ are used to depict the innovative functional connections between cities. There are much differences the three theoretical schools in the characteristics of innovation network: The new regionalism school with industrial cluster and regional innovation system as the theoretical core focuses on local embeddedness and regional knowledge spillover based on local network^15^; The Global innovation network school, with global production network and global value chain as the core theory, attaches importance to the network rights of transnational corporations and global knowledge acquisition through cross-border networks^16^; The relational economic geography school puts forward the theory model of “buzz-and-pipeline” and emphasizes the coupling of “local buzz-global pipelines” knowledge network at different spatial scales^17^. The formation process of innovation network is guided by the combined internal dynamics and external dynamics generated by the interaction of various elements in the network. The internal dynamics include the overall scale and characteristics of the network, the overall characteristics of the network, the embeddedness, the externality, absorptive capacity, the small world. The external dynamics include geographical proximity, social proximity, cognitive proximity, institutional proximity, cultural proximity^18,19^.

**Figure 1:**
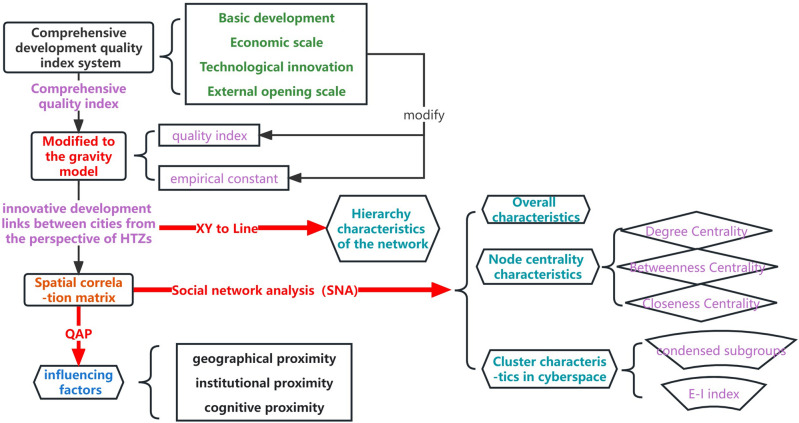
Overall methodology framework.

In recent years, the rise of the network paradigm has sparked an increasing interest and recognition in comprehending the structures of innovation networks in the realm of spatial analyses^20^. But research on the inter-city innovation correlation network based on HTZs does not draw much emphasis. There are few related literature described the function of association network among HTZs. We contribute to the literature by taking the perspective of HTZs into account when examining the innovation network structures of Chinese cities, this aims to offer novel insights for enhancing the innovation capabilities of HTZs, with the goal of accelerating the development of a high-quality national innovation system. Based upon an analysis of the knowledge of innovation network, our study focuses on 142 cities nationwide as our research subjects. We utilize attribute data from high-tech zones to construct a comprehensive development quality index system. Employing the entropy method, we calculate the quality index, which in turn modifies the gravity model to derive the innovation spatial correlation matrix. Integrated with social network analysis techniques, our exploration encompasses three key indicators: network density, average path length, and average clustering coefficient. Additionally, we delve into node centrality characteristics through metrics such as degree centrality, proximity centrality, and betweenness centrality. We then utilize methods like cohesive subgroup analysis and the *E-I* index to delve into the spatial clustering traits of the innovation association network. For an in-depth understanding of the influence mechanism behind innovation association, we employ the QAP analysis method. Our comprehensive methodology framework, depicted in Fig. 1, guides the entirety of our research endeavor. The results indicate that we constructed an inter-city innovation correlation network based on HTZs by computing network characteristics to uncover relationships between cities. The paper is organized as follows. The next section presents the empirical analysis, while Section “Discussion” provides a detailed discussion of findings, conclusions and perspectives for future research. Section “Methods” outlines the methodology.

## Results

### Overall characteristics of the network

The Density of urban innovation associated network is measured by density tool of software Ucinet. The results show that the overall connection of Chinese urban innovation correlation network is not strong, and the connection efficiency is low. The network density value is 0.084. Compared with the results of Jiamin Liu's finding^21^, the overall network density is low, which reflects the lower degree of innovative connectivity between cities. The software Gephi was employed to measure the average path length and the average clustering coefficient, yielding values of 3.083 and 0.630, respectively. A numerical value of 3.083 for the average path length is indicated that each city needs an average of three intermediary cities to realize the connection. Notably, when compared to other similar-scale innovative network research^22^, our study reveals that the average clustering coefficient (0.630) is significantly higher than that observed in China's innovation network based on patent transfers (0.420). It becomes evident that there is a substantial surplus of redundant innovation contact channels in the innovation network based on the perspective of the HTZs, and the accessibility of the overall innovation network is poor.

### Hierarchy characteristics of the network

**Figure 2:**
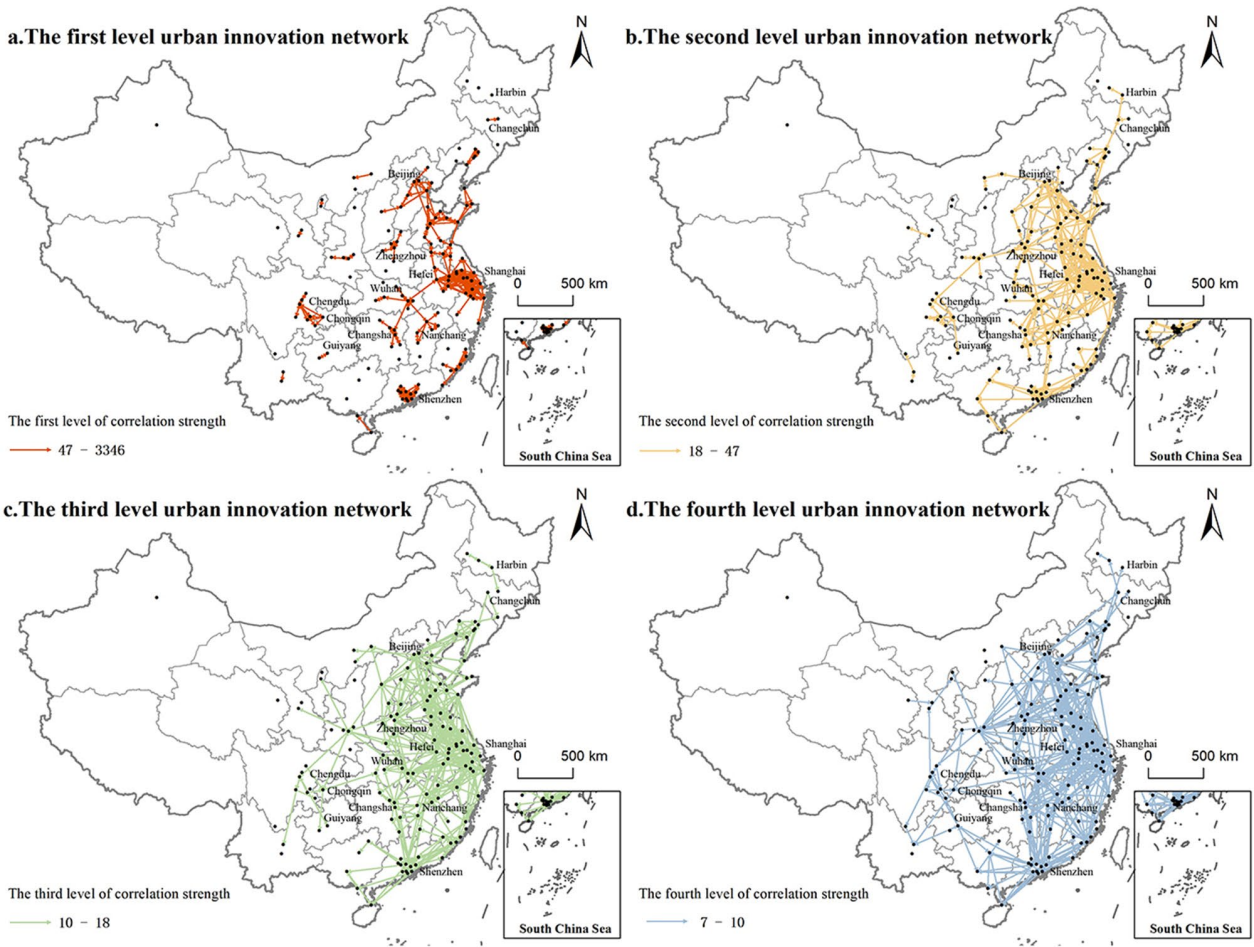
The hierarchies for city-based network in China based on innovation association. (This map is drawn by ArcGIS10.2(URL: https://developers.arcgis.com/) based on the standard map No. GS(2019)1822 approved by the Chinese Ministry of Natural Resources. The base map is not modified and does not contain data of Hong Kong, Macao and Taiwan of China. The same below).

Based on the strength value of innovation connection, the truncation threshold is determined by calculating the overall mean value. Innovation connections with strength values surpassing this mean are regarded as the foundation for identifying instances of innovation correlation between cities. And then, XY to Line operation in ArcGIS10.2 was harnessed to establish linkages of city-dyad. To visually represent the data, a quantile method was subsequently applied to categorize the strength values into four distinct levels (as depicted in Fig. 2). The overall spatial pattern of the four-tier innovation correlation network with “Z” as the main axis is presented.

The first-level connection strength threshold is set at (47, 3346). Within this first-level network, the central city assumes the role of a trailblazer in shaping the innovation connectivity landscape and constructing the framework for innovation connections across the urban agglomeration. A significant portion of these central cities are comprised of municipalities, provincial capitals, or sub-provincial cities, endowed with distinct policy environments and institutional advantages. Anchored by the core hubs of Beijing, Shanghai, and Shenzhen, innovative linkages flourish within the Beijing–Tianjin–Hebei region, Yangtze River Delta, and Pearl River Delta city clusters, respectively. Nevertheless, disparities emerge among these three regions. Urban innovation connectivity across the Beijing-Tianjin-Hebei region exhibits a scattered pattern. In contrast, the Pearl River Delta region showcases a denser urban innovation network, facilitated by its compact urban layout. The Yangtze River Delta region demonstrates a pattern of innovative and collaborative development involving three provinces and one city. Furthermore, the first-level network encompasses innovation connections between the urban agglomeration in the middle reaches of the Yangtze River (such as Wuhan → Changsha and Wuhan → Nanchang) as well as within the Chengdu-Chongqing economic circle (notably Chongqing → Chengdu).

The second-level connection strength threshold is set at (18, 47), encompassing connections that extend beyond the primary level, primarily towards the eastern and central regions. Alongside the ongoing expansion of their influence around provincial capital cities, a noteworthy trend emerges: innovative linkages between cities spanning provincial boundaries are beginning to take shape. Notably, the Hubei, Jiangxi, and Hunan regions are progressively establishing connections with cities in the eastern region (e.g., Wuhan → Zhengzhou, Hefei → Nanchang). This development, in tandem with the connections established within the Beijing-Tianjin-Hebei region and the Yangtze River Delta region, forms the foundation of an “Z-shaped” eastern triangle connection framework. Furthermore, there is an additional facet to this landscape. Innovative connectivity is observed linking cities in the southwest with Guizhou (notably Chongqing → Guiyang), while a distinct network emerges connecting cities in the Northeast (Changchun → Harbin).

The third-level connection strength threshold is set at (21, 40). Within the third-level network's eastern region, the urban innovation network connections exhibit a gradual evolution toward complexity and density, culminating in the formation of a distinct “Z-shaped” network pattern. The connections observed in this eastern region assume a combination of overall dispersion and localized agglomeration. In stark contrast to the sparsely connected innovation landscape prevalent in the western region, the core cities within the western sector display diminished radiation influence, characterized by loosely interwoven and isolated innovation connections. Consequently, the structural framework of the innovation correlation network from the perspective of the HTZs has yet to crystallize in the western region. With Shanghai and Shenzhen as pivotal vertices, the “Z” shaped southern triangle firmly establishes itself throughout the Yangtze River Delta, Pearl River Delta, and the middle reaches of the Yangtze River, marking a significant advancement in the network's spatial configuration.

The fourth-level connection strength threshold is set at (7, 10). This fourth-level network stands as the foundational framework for China's urban innovation correlation network, presenting a striking spatial arrangement akin to a lightning bolt, with the “Z” shape as its central axis. Unlike the preceding three levels of urban innovation connections that primarily hinge on geographical proximity among central cities, the fourth-level network showcases the lowest connection intensity yet the broadest scale. It predominantly comprises long-distance, non-proximity connections involving marginal cities. Within this fourth-level network, the “Z”-shaped pattern, anchored by the Beijing-Tianjin-Hebei region, the Yangtze River Delta, the middle reaches of the Yangtze River, and the Pearl River Delta, reveals more densely woven innovation connections among nodes within these core regions compared to those outside. However, the overall network support in the Chengdu–Chongqing region is lacking, and the high-level innovation connections with the middle reaches of the Yangtze River and the Pearl River Delta urban agglomerations remain limited. Consequently, the overall structural configuration falls short of realizing the envisioned “diamond” pattern within the network.

### Node centrality characteristics

Degree Centrality, Betweenness Centrality, and Closeness Centrality are quantified using the Centrality tool within the Ucinet software. The results underscore that the degree centrality within the eastern region surpasses that in the western counterpart. Notably, the Betweenness Centrality of core cities situated in central China assumes a pivotal bridging role, effectively connecting various components of the network. Furthermore, cities positioned in outlying areas in proximity to the central core tend to exhibit heightened susceptibility to the influence exerted by interconnected cities.

In terms of Degree Centrality, cities in eastern regions consistently hold higher positions compared to their counterparts in the western regions. The ranking of Degree Centrality for Beijing and Shanghai underscores their central role within the network, as they exhibit the strongest associations with other cities, thereby wielding a substantial influence over the innovation efficiency of those cities. Notably, Hefei, Nanjing, Wuhan, and several other cities display higher point-in degrees in comparison to their point-out degrees, signifying the palpable spillover effect originating from these cities. However, a distinct pattern is evident in cities like Zaozhuang, Suqian, and Xuzhou, where their point-in degrees are lower than their point-out degrees. This discrepancy suggests that these cities possess weaker external radiation capacity in comparison to their capacity to receive external influences.

In terms of Betweenness Centrality, the core cities within central China emerge as vital intermediaries. Wuhan, Hefei, and Xi’an, among other central urban centers, distinctly stand out as the top three intermediary hubs, significantly surpassing other cities in this regard. This observation underscores the pivotal status of these cities within the regional innovation connectivity network. Acting as crucial “bridges” within the network, they exert substantial control over the innovation connections between cities.Furthermore, it's noteworthy that cities such as Xining, Qiqihar, and Yuxi exhibit an intermediary center degree value of 0. Positioned in more remote areas, these cities have yet to assume an intermediary role within the network.

In terms of Closeness Centrality, the ranking diverges from that of Degree Centrality and Betweenness Centrality. Notably, Nanchang, Hangzhou, and Zhengzhou are positioned prominently in terms of Closeness Centrality. These cities exhibit short shortcut distances to other urban nodes within the innovation connection network, facilitating the efficient transfer of innovation elements.Conversely, Urumqi, Yulin, and Ankang attain lower rankings in Closeness Centrality, indicating longer shortcut distances from other cities within the network. Consequently, the transfer of innovative elements becomes more challenging for these cities. Shanghai and Beijing emerge as leaders in inner Closeness Centrality, wielding significant influence over other regions. This influence can be attributed to their favorable innovation-oriented geographical contexts. Moreover, Yinchuan and Shizuishan top the charts in terms of outer Closeness Centrality. This observation underscores that these regions are significantly influenced by other cities. This phenomenon is a direct result of remote areas featuring fewer innovation correlations, and their innovation resources predominantly emanate from neighboring cities, rendering them more susceptible to the influence of related urban nodes.

### Spatial clustering characteristics of networks

To analyze the spatial clustering features, we initially employ the Concor Algorithm (Iterative Correlation Convergence) within the Ucinet software for cluster analysis of the urban innovation correlation network. This analysis effectively partitions the urban innovation correlation network into 8 distinct third-level subgroups and 4 secondary-level subgroups. The latter four subgroups further form innovation aggregation subgroups, delineated as the Northern Hinterland, Eastern Coastal, Southern Region, and Western Cooperative subgroups. By computing the average innovation quality index and centrality metrics for each innovation concentration subgroup (as depicted in Table 1), several observations come to light. Notably, the Northern Hinterland and Southern Region subgroups comprise a substantial number of cities. The Eastern Coastal subgroup, on the other hand, stands out with the highest centrality ranking among the subgroups, underscoring its significance in the network. In contrast, the Western Cooperative subgroup lags behind in terms of centrality metrics.

**Table 1:** Intercity cooperative innovation innovation network clusters.

Innovate condensed subgroups		North hinterland subgroup	Eastern coastal subgroup	Southern region subgroup	Western cooperative subgroup
Degree Centrality C_*D*_	Point-in degree	10.13	23.19	11.09	4.24
	Point-out degree	9.82	24.12	10.65	4.64
	Centrality	19.96	47.31	21.74	8.88
Betweenness Centrality C_*C*_		22.85	29.43	25.43	15.73
Closeness Centrality C_*B*_	The inner Closeness Centrality	7.74	8.02	7.79	7.80
	The outer Closeness Centrality	30.59	37.45	33.21	23.52
	Centrality	279.06	285.89	259.98	263.16
E-I index		0.161	0.083	0.350	− 0.199
Number of nodes		45	26	46	25
Innovation quality index		1447.29	2051.14	1623.65	1498.20

In terms of city distribution, the Northern Hinterland and Southern Regions encompass the greatest number of cities. Conversely, the Eastern Coast and Western Collaboration subgroups are comparatively smaller, accounting for only 36% of the total city count. When considering innovation quality, cities within the Eastern Coastal subgroups exhibit a notably elevated average innovation quality index of 2051.14, surpassing that of the other three subgroups. This divergence can be attributed to the concentration of universities and scientific research institutions in eastern China, fostering distinct talent advantages and innovative resource endowments. Turning to centrality, the Eastern Coastal subgroup showcases an average Degree Centrality of 47.31, a figure nearly five times higher than the average degree for the Western Cooperative subgroup, which holds the lowest rank. The Degree Centrality values for the Northern Hinterland and Southern Region subgroups are relatively comparable. Remarkably, the Eastern Coastal subgroup displays a point-in degree exceeding its point-out degree, indicative of a pronounced innovation “spillover effect.” Conversely, the Northern Hinterland and Southern Region subgroups manifest a point-in degree surpassing their point-out degree, giving rise to a “siphon effect” that channels numerous innovation factors such as knowledge, technology, and talent out of the Western region. In terms of intermediation, the Eastern Coastal and Southern subgroups exhibit elevated intermediary centrality, attributable to the well-established economic foundations and accessible high-speed rail networks within the Yangtze Valley and South China. These factors confer superior geographic bridging advantages, facilitating connections among cities in other subgroups. Moreover, both the Eastern Coastal and Northern Hinterland subgroups display heightened Closeness Centrality. This results in a strong attractive force and influence over cities in other regions, thereby fostering a broad range of innovation radiation. Consequently, these subgroups can efficiently establish direct connections with inner cities, expediting the dissemination of innovation factors like knowledge and technology.

**Figure 3:**
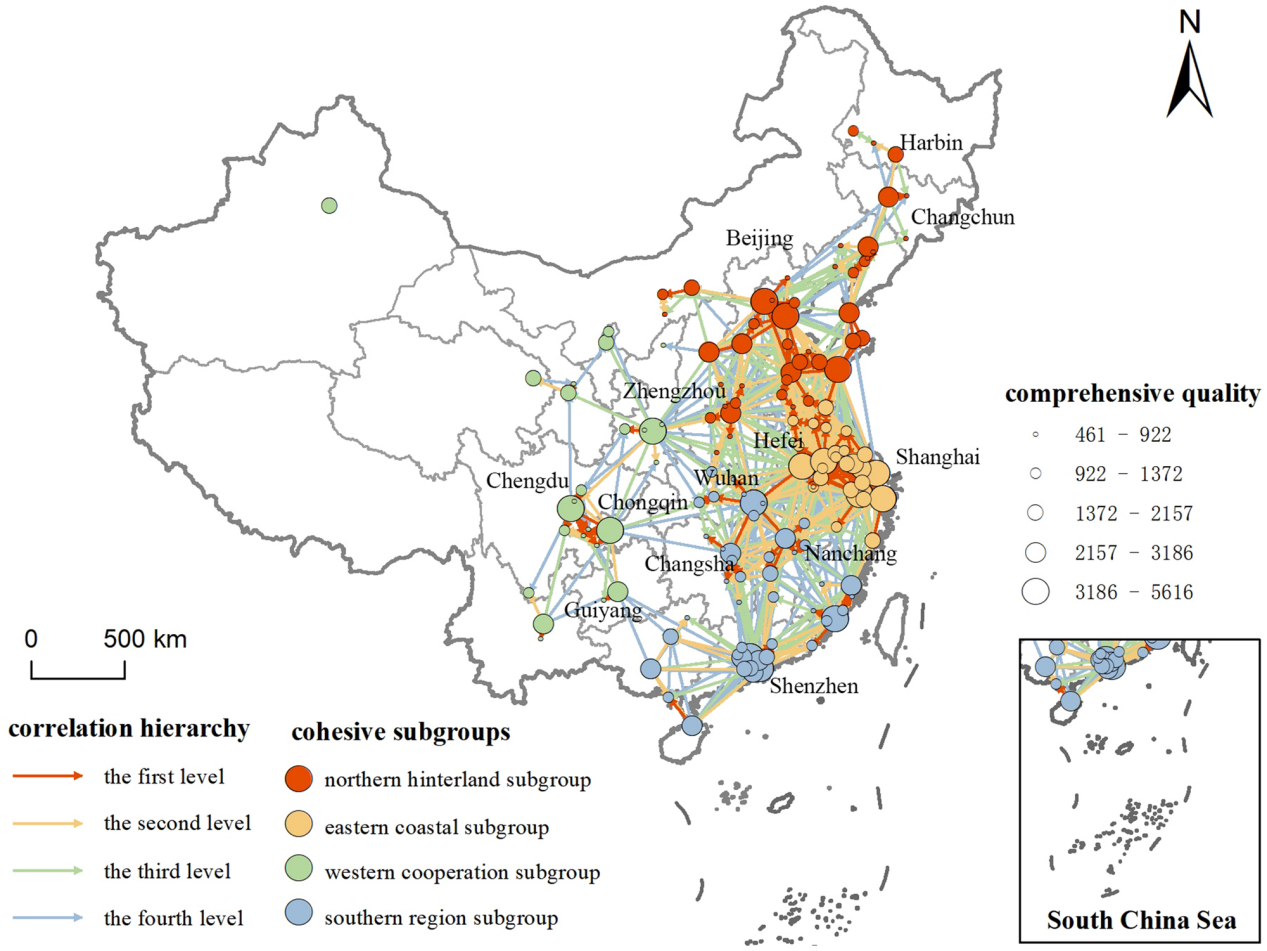
The structure of intercity cooperative innovation network in China.

Furthermore, the Cohesion tool within the Ucinet software was employed to assess the internal *E-I* index of each subgroup, shedding light on their internal clustering characteristics (as depicted in Fig. 3). The findings illuminate distinct patterns: the Northern Hinterland, Eastern Coastal, and Southern Regions subgroups all exhibit small group network phenomena, though these network characteristics aren’t overtly pronounced. In this context, the Eastern Coastal subgroup assumes the role of a “central actor,” while the Western Cooperative subgroup assumes a more “marginal actor” position. The *E-I* index of the Western Cooperative subgroup approaches -1, signaling that the intra-group urban network density exceeds that outside the subgroup. This phenomenon arises from the absence of cities wielding strong radiation and driving effects, making it challenging to establish dominant connections with other cities. As a result, this subgroup maintains a degree of independence from the broader innovation network. Conversely, the Northern Hinterland, Eastern Coastal, and Southern Regions subgroups all exhibit positive *E-I* indices. However, the Northern Hinterland and Eastern Coastal subgroups display lower absolute *E-I* index, suggesting that their subgroup innovation connections are primarily self-derived. Although the *E-I* indices of these two innovation condensation subgroups hover near 0, the reasons are distinct. The Eastern Coastal subgroup's *E-I* index approximates 0, indicating that the urban network's connection density within the region closely mirrors its external counterpart. This phenomenon is driven by Shanghai's robust core city radiation, facilitated by the efficient Yangtze River Delta urban agglomeration transportation network. Administrative constraints play a role in diminishing boundary effects, resulting in better integration of the regional network into the broader innovation network. Comparatively, the Northern Hinterland subgroup’s *E-I* index slightly surpasses that of the Eastern Coastal subgroup, signifying a more pronounced small group phenomenon within the Beijing-Tianjin-Hebei region. Simultaneously, a stronger trend of innovation and integration emerges in the Yangtze River Delta urban agglomeration. The Southern Region subgroup boasts an *E-I* index closest to 1. Notably distinct from other subgroups, its innovative association network connections extend beyond the region, with internal network density trailing behind the external counterpart. This dynamic is fueled by the proximity of Hong Kong, Shenzhen, and Guangzhou, promoting the interplay of inter-city innovation resources and connections within the Pearl River Delta urban agglomeration.

### Analysis of the influencing factors

The QAP correlation analysis of the correlation matrix of urban innovation in China was conducted by Ucinet Software. The analysis involved 10,000 random substitutions to derive the correlation coefficient and significance levels between the dependent variable matrix and various influencing factors (as detailed in Table 2). The outcomes demonstrated that the correlation coefficients for spatial neighbor relationships, government administrative levels, openness to the outside world, and differences in science and technology investment passed the rigorous 1% significance level test. Furthermore, the difference in innovation output surpassed the 5% significance level test. These results indicate a substantial and statistically significant correlation between the aforementioned independent variables and the inter-city innovation associations. However, it's worth noting that the disparities in industrial structure and economic development did not meet the significance criteria in the test. This preliminary analysis suggests that differences in industrial structure and economic development may not exert a significant influence on the establishment of inter-city innovation correlations. According to the regression results, the spatial proximity relationship, administrative rank difference, industrial structure difference and opening to the outside world difference passed the significance level test of 1%, and the standardized regression coefficient of spatial proximity relationship was much higher than other factors. The difference in science and technology investment passes the significance level test of 5%, and the difference in economic level passes the significance level test of 10%.

**Table 2:** Urban innovation a ssociation network of China QAP analysis results.

Argument	Dependent variable IA city innovation association matrix			
	QAP correlation	QAP regression	80% QAP regression	120% QAP regression
*Geo*	0.419***	0.412***	0.378***	0.437***
*Ins*	− 0.079***	− 0.110***	− 0.122***	− 0.101***
*Ind*	− 0.003	− 0.042***	− 0.051***	− 0.039**
*Eco*	0.013	0.023*	0.023*	0.025**
*Ext*	0.106***	0.068***	0.072***	0.070***
*Sci*	0.078***	0.042**	0.048**	0.035**
*Inn*	0.036**	0.008	0.012	0.075
R^2^		0.193	0.170	0.213
Adj-R^2^		0.192	0.169	0.212

***Means p-value < 0, **means *p*-value < 0.01, and *means *p*-value < 0.05.

*p*-value(probability value), *p*-value < 0.05 is considered to have a statistical difference, and *p*-value < 0.01 is considered to have a significant statistical difference.

Through a multidimensional proximity lens, the regression coefficient of spatial proximity (*Geo*) emerges with a substantial positive absolute value, underscoring the role of geographical proximity in fostering urban innovation associations. Spatial adjacency serves to alleviate spatial barriers in innovation communication and dilute the positive externalities of knowledge spillover. In the case of cities within the Western Cooperative subgroup, which are widely dispersed, the transmission of innovation elements and technological exchange faces heightened distance friction, hampering the establishment of robust innovation connections. Conversely, the coefficient for administrative rank difference (*Ins*) exhibits a significant negative trend, indicating a negative correlation between institutional proximity and innovation cooperation. This phenomenon arises from local protectionism, which generates institutional costs in enterprise-driven innovation cooperation, ultimately impeding cross-regional innovation linkages. For instance, consider the cases of Daqing in Heilongjiang Province and Tonghua in Jilin Province, both general cities in terms of administrative levels. While these cities share industries like petrochemicals and pharmaceuticals, inter-provincial administrative barriers hinder the development of strong innovation connections, even when industries exhibit high levels of correlation. Regarding the coefficient for industrial structure difference (*Ind*), a notably negative value emerges, signifying that cities with a similar perceived proximity demonstrate a heightened likelihood of forming innovation linkages. Strong innovation connections are more likely to flourish between cities boasting similar economic strengths and industrial structures. However, cities like Changzhi and Ordos, primarily reliant on the secondary industry, face challenges in swiftly cultivating an innovation-friendly industrial environment. Consequently, forging robust innovation connections with cities like Shanghai, Guangzhou, and Shenzhen, which predominantly embrace the tertiary industry, remains difficult in the short term.

From an economic and technological innovation perspective, the coefficient for economic level difference (*Eco*) demonstrates a positive link with urban innovation associations, while the disparity in openness to the outside world (*Ext*) is positively correlated with the dependent variable matrix. Variations in economic development and degrees of openness give rise to diverse pricing for innovation factors across cities. Typically, economically developed coastal regions command higher innovation factor prices, which in turn prompts the flow of these factors from low marginal return coastal cities to high marginal return inland cities. This dynamic spurs technological interactions and knowledge dissemination between cities. Meanwhile, the science and technology investment difference (*Sci*) coefficient appears positive. This finding suggests that differences in science and technology investment facilitate the establishment of innovation connections between cities. Notably, central cities often boast the highest science and technology investment levels in their respective regions. Through the “siphon effect” generated by surrounding cities, these central cities play a pivotal role in engendering robust innovation connections within their regions. A case in point is Wuhan and Nanchang, the central cities within the urban agglomeration of the Middle reaches of the Yangtze River. Here, the proportion of science and technology expenditure in their public budgets is about two to three times higher than that of surrounding general cities. This significant investment acts as a magnet for neighboring regions, encouraging them to engage in innovative exchanges and interactions. However, the innovation output difference (*Inn*) did not meet the significance test, implying that the number of granted patents index has no discernible impact on the establishment of innovation correlations.

To further validate the robustness of the QAP regression outcomes, a technique inspired by Sun Zhongrui's research^23^ was employed. This involved selecting 80% and 120% of the mean value of the dependent variable within the urban innovation correlation matrix as thresholds. Different binarization matrices for urban innovation correlations were consequently constructed, serving as new dependent variable matrices. Subsequently, QAP regression analyses were conducted employing the same set of seven independent variable matrices as before. This robustness test aimed to assess the stability of the results.The investigation revealed that neither the coefficients nor the significance levels of the same independent variable matrix exhibited noteworthy changes under the two thresholds. This consistency underscores that the factor matrix effectively explains the variation in the independent variable matrix. As a result, the regression outcomes exhibit heightened robustness and credibility.

## Discussion

This study delves into the urban innovation correlation through the lens of HTZs, offering a novel perspective that enriches the understanding of urban innovation networks. This approach expands the horizons of urban and economic geography by examining development zones and regional innovation, particularly in the context of the Chinese urban innovation correlation network from the HTZ perspective. Distinct from other urban networks, this perspective carries significant practical implications, particularly for establishing connectivity channels between high-tech enterprises and crafting regional innovation-driven development strategies. Consequently, the urban innovation network, fostered through HTZ linkages, should possess the capacity to effectively facilitate the movement of intercity innovation factors such as talent, technology, and capital. Moreover, this network is poised to exert a positive influence on the national innovation system and even the global innovation ecosystem. Drawing upon prior research on innovation network influence mechanisms, several strategies can be considered for further development: ① Elevate the Global Integration of the Eastern Coastal Subgroup: By establishing overseas innovation centers, international business bases, and cooperation parks, the Eastern Coastal Subgroup can deepen collaboration with countries along the “Belt and Road.” This approach enables talent and technology exchanges, fostering cross-border cooperation. Such efforts would augment the linkages between domestic HTZs and international innovation hubs, facilitating the influx of high-end global innovation resources. This alignment would further integrate global supply chains and industry pipelines. ② Overcome Administrative Barriers in the Western Cooperative Subgroup: Alleviating regional constraints by distributing innovation factors can invigorate market players and bolster the synergistic development of regional economies. This approach promotes the creation of collaborative and complementary innovation communities, driving comprehensive progress in economy and technology. ③ Enhance Collaboration and Exchange within Subgroups: Strengthening collaboration and exchange between different subgroups, such as the Northern Hinterland and Southern Subgroup, or the Western Cooperative Subgroup and Eastern Coastal Subgroup, can lead to mutual benefits. This can be achieved through various mechanisms such as remote incubation, enclave economies, and partner parks. Such endeavors can bridge the gap between regions and establish platforms for knowledge and resource sharing.

Our findings underscore a network that, on the whole, lacks close connectivity, suffers from a high number of redundant channels, and exhibits low efficiency, resulting in poor network accessibility. The hierarchical structure unveils a lightning-like spatial pattern dominated by a “Z-shape” configuration, with Beijing, Shanghai, and Shenzhen standing as the core hubs. Specifically, the first-level network is led by central cities, establishing the framework of urban agglomerations. The second-level network extends connections between the eastern and middle regions. In the third-level network, connections in the eastern part become increasingly intricate. The fourth-level network encompasses more marginal cities. Our contribution to the innovation network literature is marked by the rigor of our social network analysis and the comprehensive examination of connectedness using various network centrality measures. Our results also highlight the disparities between urban areas in the eastern and western regions. The Eastern region's Degree Centrality surpasses that of the Western region, with Beijing and Shanghai emerging as frontrunners. Cities like Nanjing exhibit pronounced spillover effects. Notably, central core cities play an intermediary role, with urban centers such as Wuhan serving as vital “bridges” within the network. In contrast, cities in remote areas face susceptibility to the influence of related cities, making it challenging for locales like Urumqi to transfer innovative elements effectively. The cluster analysis yields significant insights, revealing four cohesive innovation subgroups: the Northern Hinterland Subgroup, Eastern Coastal Subgroup, Southern Region Subgroup, and Western Cooperative Subgroup. Among these, the first three subgroups exhibit characteristics of small group networks. The Northern Hinterland and Southern Region Subgroups encompass a larger number of cities. The Eastern Coastal Subgroup boasts the highest central degree, positioning it as the “central actor,” while the Western Cooperative Subgroup assumes a “marginal actor” role. Turning to the influencing factors of city-based innovation correlations in China, our results present a mixed picture. Spatial proximity emerges as the primary driver of urban innovation connections. Factors such as differences in administrative rank, economic development level, openness degree, and science and technology investment contribute to innovation connections between cities. Simultaneously, similarity in spatial proximity and industrial structure enhances strong innovation connections between cities.

The research findings unveil that the Chinese urban High-Tech Zones (HTZs) have formed a network, albeit one that is not thoroughly connected and efficient. Consequently, there is a necessity for the urban innovation network to effectively leverage the diverse comparative advantages inherent in regional agglomeration subgroups. This approach will facilitate the efficient aggregation and rational flow of innovation factors, thereby promoting a path towards modern, high-quality innovation development networks. However, it's important to acknowledge certain limitations and areas that warrant further exploration. Primarily, the intricate structure of the urban innovation network presents challenges in accurately defining and quantifying innovation relationships between cities, especially when utilizing models to indirectly simulate correlation networks. Additionally, constructing an innovation network solely based on specific attribute data of HTZs overlooks the constraining impact of the inherent economic development framework on high-tech industries and the innovation research and development within HTZs.Furthermore, the study on the mechanism of multidimensional proximity within innovation networks focuses on the regression analysis of proximity's impact on innovation networks. The dynamic interplay between multidimensional proximity and the evolution of innovation networks isn't thoroughly discussed. Hence, it's imperative to delve deeper into understanding the evolution characteristics of innovation networks across various spatial and temporal dimensions. Further research endeavors should explore the combined mechanisms of multidimensional proximity in a comparative analysis, contributing to a more comprehensive understanding of this intricate dynamic.

## Methods

### Association relationship determination

By considering cities as nodes and the intensity of innovation connections between them as connections within the network, the urban innovation correlation network in China is depicted. This network is formed through the interplay of points and lines, illustrating the relationships between cities. The establishment of this network relies on comprehensive data, encompassing 16 secondary indexes that constitute the High-Tech Zones (HTZs) quality index system for comprehensive development. These indexes are categorized under basic development, economic scale, technological innovation, and external opening dimensions (as outlined in Table 3). Beginning with the comprehensive development of HTZs, a profound link exists with urban infrastructure development^24^. On the city level, fundamental HTZ development is represented by indicators such as urban administration, social security, investment in science and education, ecological environment, and industrial pollution control. Moving on to the HTZs level, the economic scale is predominantly gauged by indicators like land use, enterprise profitability and management, and contribution to the regional economy^25^. Addressing technological innovation, factors encompass the status of high-tech enterprise development, as well as manpower and financial inputs. Typically, high-tech enterprises tend to establish themselves in regions marked by substantial research and development expenditures, alongside significant investments in human capital^26^. This strategic placement facilitates access to novel technologies and fosters an environment conducive to productive market interactions. Lastly, external opening scale aspects incorporate elements such as enterprise export volume within HTZs and the presence of international talent. These factors gauge the competitive edge HTZs possess within the global market. Utilizing the entropy method, the comprehensive development quality index for HTZs is measured. This index reflects the quality of urban innovation, thereby enabling adjustments to single-factor quality indexes and empirical constants within the traditional gravity model. The distance between cities is represented by the shortest driving distance between two HTZs. Consequently, the revised gravity model takes the following form:

1 $$ \left\{ {\begin{array}{*{20}l} {f_{ij} = k_{ij} \frac{{M_{i} M_{j} }}{{d_{ij}^{2} }}} \hfill \\ {k_{ij} = \frac{{M_{i} }}{{M{}_{i} + M_{j} }}} \hfill \\ \end{array} } \right. $$

In formula: f_*ij*_ represents the innovation connection strength value between cities *i* and *j*, M_*i*_, M_*j*_ respectively represent the innovation quality index of city *i* and *j*, d, represents the distance of the city *i*, *j*, k_*ij*_ represents the modified empirical constant of the proportion of the innovation quality index of city *i* in the sum of the innovation quality index of city* i* and *j*.

**Table 3:** Comprehensived evelopment quality index system of high tech zone.

Dimension	Factor	Quantitative	Meaning
Basic development	X1	Municipalities are assigned a value of 3, provincial capitals and sub-provincial cities are assigned a value of 2 and others are assigned a value of 1	Urban administrative level
	X2	(Endowment insurance + medical insurance + unemployment insurance) Participation rate/3 (%)	Urban social security
	X3	Science and technology expenditure/local general budget expenditure of prefecture (%)	The importance of urban science and technology
	X4	Centralized treatment rate of sewage treatment plant is (%)	Urban ecological protection, environmental protection and green development
	X5	Investment of the industrial pollution control project of the local province was completed this year (1000 RMB)	Emphasis on urban industrial pollution control
Economic scale	X6	GDP of HTZS/approved area (hm^2^)	Unit land use intensity and economic benefits of HTZs
	X7	Net profit/operating income of enterprises in the HTZ (%)	Enterprise profitability and development performance of HTZs
	X8	Number of enterprises integrated in the HTZ/number of industrial and commercial registered enterprises is (%)	Enterprise management and cultivation in HTZs
	X9	The industrial GDP/GDP of the urban area is (%)	The contribution of HTZs to urban economy and industry
Technological innovation	X10	The Number of high-tech enterprises/the number of enterprises registered in the information database is (%)	Development status of high-tech enterprises
	X11	Full-time equivalent of R&D personnel of HTZ enterprises/ending number of employees (%)	Manpower input for independent innovation in HTZs
	X12	Internal expenditure of R & D funds in HTZ/enterprise operating income (%)	Investment in R&D and technological innovation in HTZs
	X13	Number of students in ordinary undergraduate and junior college in the city (people)	Comprehensive quality of the employees in HTZs
External opening scale	X14	Total export amount of enterprises in the HTZ (one thousand RMB)	The degree of opening up the outside world of HTZs
	X15	Export volume/operating income of enterprises in the HTZs (%)	The ability of HTZs to export to international competition
	X16	(Number of foreign resident + returned students studying abroad)/The final number of employees: (%)	The internationalization degree of the employees in the HTZ

Based on the strength value of urban innovation connection, we construct the innovation correlation matrix. Taking the 142 cities in China as nodes and the innovation connection strength as the edges, we implement a binarization procedure. Specifically, we utilize the overall mean value as the truncation threshold. Connections with values greater than the mean are designated as 1, while those below the mean are assigned a value of 0. This binary approach effectively indicates the presence or absence of innovation correlation between cities. Consequently, this transforms the numerical matrix into a directed symmetric matrix, capturing the essence of the interconnections.

### Social network analysis

We examine the overarching characteristics of the urban innovation correlation network through the lens of three key indicators: network density, average path length, and average clustering coefficient (as presented in Table 4).The network density index serves as a metric to gauge the spatial correlation of innovation within the network. A higher network density indicates a more intimate innovation correlation among the cities in the network, highlighting a stronger interconnectedness. On the other hand, the average path length is a measure of the average shortest path between any two nodes. It offers insights into network accessibility and the typical distance between nodes. A smaller average path length signifies that the connection between any two nodes requires fewer intermediary steps, thus implying better network connectivity and accessibility^27^. Furthermore, the average clustering coefficient quantifies the likelihood of interconnection between two nodes that are connected to the same central node. This metric effectively evaluates the tightness of node clustering within the network, shedding light on the level of agglomeration among nodes^28^.

**Table 4:** Expression and interpretation of innovation network indicators.

Measure	Metric	Formula	Explain
Overall network indicators	Network density	$D = \frac{2r}{{n(n - 1)}}$	*n* is the number of members in the network (the same below); *r* is the actual number of relationships included in the city contact network
	Average path length	$L = \frac{2}{n(n - 1)}\sum {d_{ij} }$	*D*_*ij*_ is the shortest distance between city *i* and city *j*
	Mean clustering coefficient	$C = \frac{1}{n}\sum {C_{i} = } \frac{1}{n}\sum {\frac{{2E_{i} }}{{k_{i} (k_{i} - 1)}}}$	*C*_*i*_ is the ratio of the actual number and the theoretical maximum number between all adjacent cities; E_*i*_ is the actual number of edges exists in the LAN formed with adjacent cities
Individual network indicators	Degree centrality	$C_{D} = \frac{l}{n - 1}$	The *l* is the number of other cities in the network directly related to a city;
	Betweenness Centrality	$C_{B} = \frac{{2\sum\limits_{j}^{n} {\sum\limits_{k}^{n} {b_{jk} (i)} } }}{{n^{{2}} - 3n + 2}}$	*b*_*jk*_ is the probability that the city *i* is on the shortcut between *jk*
	Closeness Centrality	$C_{c} = \sum\limits_{j = 1}^{n} {d_{ij} }$	*D*_*ij*_ is the shortcut distance between city *i* and city *j*
Network condensed subgroups	*E-I index number*	$$\begin{aligned} E - I = & \frac{{\rho_{EL} - \rho_{IL} }}{{\rho_{EL} + \rho_{IL} }} \\ \rho_{EL} = & {{eRC_{i} } \mathord{\left/ {\vphantom {{eRC_{i} } {\left( {(n - k)(n - k - 1)/2} \right)}}} \right. \kern-0pt} {\left( {(n - k)(n - k - 1)/2} \right)}} \\ \rho_{IL} = & {{iRC_{i} } \mathord{\left/ {\vphantom {{iRC_{i} } {\left( {k(k - 1)/2} \right)}}} \right. \kern-0pt} {\left( {k(k - 1)/2} \right)}} \\ \end{aligned}$$	*eRC*_*i*_ is total amount of regional external contact; iRC_*i*_ is the total connection amount within the region;*ρ*_*EL*_ is out-of-region network density; *ρ*_*IL*_ is the network density outside the region; *k* is the number of city nodes in the region

Utilizing metrics such as Degree Centrality, Betweenness Centrality, and Closeness Centrality, our study delves into the centrality characteristics of nodes within the innovation correlation network. Through this analysis, we uncover the positions and roles of each node within the broader innovation correlation network. Degree Centrality measures the number of direct connections a city has with other cities. A high Degree Centrality implies that the city occupies a central position within the innovation correlation network, showcasing a robust capacity to receive external influence. The point-in degree signifies the city's beneficial effect, while the point-out degree indicates its spillover effect on other cities. Moving to Betweenness Centrality, it reflects a city's control over innovation elements. A higher Betweenness Centrality indicates that the city possesses a stronger bridging capacity, acting as a key intermediary between other nodes in the innovation correlation network. Closeness Centrality quantifies the sum of shortcut distances between a city and all other cities. A smaller value corresponds to higher Closeness Centrality, highlighting strong innovation mobility between cities. Inner Closeness Centrality underscores a city's ability to influence others, whereas outer Closeness Centrality showcases the degree of influence exerted by other cities on it^29^.

To delve into the clustering characteristics of the innovative relational network space, we employed the condensed subgroups clustering method alongside the *E-I* index. Cohesive subgroups refer to closely interconnected urban subsets, serving to unveil and describe the internal substructure of smaller to medium-sized groups within the innovation correlation network. This approach sheds light on the inner structure of these small groups. The *E-I* index is a vital tool in this analysis, indicating whether distinct small group network characteristics are evident within the innovation association networks^30^. When the *E-I* index approaches -1, it signifies that the internal network density of subgroups far surpasses that of the external network. In such cases, the members within subgroups share a strong affiliation, and these subgroups tend to function somewhat independently from the broader urban innovation network. On the other hand, an E-I index close to 0 suggests that the internal network density of the subgroup aligns with that of the external network. This indicates that the internal innovation association network of the subgroup is effectively integrated into the overall network structure. Conversely, an *E-I* index near 1 suggests that the internal subgroup network's density is notably lower than the external network's density. In this scenario, the majority of innovative connections occur outside the subgroup, illustrating the significance of external connections.

### QAP analysis

In the context of traditional regression analysis methods, the inherent autocorrelation within the relationship matrix structure can render the significance testing of variables ineffective. To overcome this challenge, we adopted the Quadratic Assignment Procedure (QAP) analysis for relationship data to delve into the factors influencing the urban innovation correlation network in China. The QAP regression analysis method, known as the Secondary Assignment Procedure, is a non-parametric test that relies on the substitution of matrix data. It is frequently used to unveil the regression relationships between a primary matrix and multiple secondary matrices, offering a way to avoid multicollinearity issues without requiring variable independence. This approach provides a more robust way to explore the complex dynamics of urban innovation correlation^31^. To further elucidate the mechanisms underpinning urban innovation correlation in China, scholars have introduced concepts such as the “local buzz-global pipelines” view and the discussion on the relationship between multidimensional proximity and innovation. The “local buzz-global pipelines” view underscores the significance of both local collaborations and long-distance knowledge exchange for fostering innovation. This approach emphasizes the interaction between proximate subjects within a region as well as the broader cross-border knowledge sharing and dissemination that facilitates innovation^32^. Incorporating the concept of multidimensional proximity, which encompasses factors like geographical, institutional, cognitive, cultural, technological, organizational, and social proximity, this research investigates how these aspects influence urban innovation^33^. However, owing to variations in research targets and spatial scopes, the impact of multidimensional proximity on innovation remains a subject of debate. In contrast to innovation subjects at the micro-enterprise level, urban-scale innovation linkages are notably influenced by factors like geographical proximity, institutional proximity, and cognitive proximity^34^.Within this context, geographical proximity underscores the spatial closeness or division between innovation subjects. Geospatial proximity facilitates industrial interactions, communication among personnel, information exchange, and collaborative efforts among innovation entities. This fosters the efficiency of technical cooperation, allowing for the seamless flow of knowledge, resources, and expertise among geographically proximate cities^35^. Institutional proximity involves the similarity of subjects under formal and informal constraints, while cognitive proximity pertains to the alignment of cognitive understandings among innovative subjects during communication^36^. Therefore, by considering multidimensional proximity alongside urban economic dynamics, openness, administrative hierarchy, industrial structure, economic development, science and technology investment, and innovation output differences, we constructed a model of seven factors as independent variables that influence the structure of the Chinese urban innovation association network. We employed the urban innovation association matrix (*IA*) as the dependent variable to further unravel the intricate influencing factors shaping the Chinese urban innovation network. Consequently, our constructed regression model took the following form:

2 $$ IA = f(Geo, \, Ins, \, Ind, \, Eco, \, Ext, \, Sci, \, Inn) $$

The independent variables in Eq. (2) are described as follows: ① Spatial adjacency (*Geo*). The geographic adjacency matrix is constructed by measuring the geographic proximity with the adjacency dummy variable (0, 1). The adjacent cities are denoted as 1, or 0 otherwise. ② Administrative rank of government (*Ins*). The administrative rank of the government is used to measure the institutional proximity between cities. If the administrative rank is the same, the value is 1, indicating that the administrative power is similar; otherwise, it is 0, so as to construct the administrative rank difference matrix. ③ Industrial Structure Differences (*Ind*). Based on the proportion of tertiary industry in gross regional product as the proxy variable of industrial structure, the difference matrix of industrial structure between cities is constructed to depict the cognitive proximity. ④ Economic Development Differences (*Eco*). The per capita GDP is used as the difference matrix of economic development and the proxy variable of economic development. ⑤ Opening to the outside world difference (*Ext*). We will measure the degree of opening up by the proportion of total imports and exports to GDP, and build a difference matrix of opening up. ⑥ Technology Investment Differences (*Sci*). The proportion of science and technology expenditure in the local general public budget expenditure represents the degree of urban support for innovation, so as to construct the difference matrix of science and technology investment. ⑦ Innovation output difference (*Inn*). The number of patents is used to represent the level of innovation output and build the innovation output difference matrix. Adopting a similar approach as outlined in Qin Qi's study^37^, we constructed the difference matrix. Initially, each unit was defined as the proportion of the independent variable attribute within the city. Subsequently, the independent variable matrix underwent binarization, with threshold values set at 1.2, 1.5, 1.2, 1.0, and 1.5 for the respective five difference matrices. This means that a value becomes 1 if it surpasses the threshold, while it remains 0 if it falls below the threshold. This series of steps ultimately yielded the binarized difference matrix of independent variables.

## Data Availability

All data generated or analyzed during this study are included in this published article. Tese datasets were derived from the following public domain resources: The basic data of the HTZs are mainly from the 2018 China Development Zone Review and Announcement Catalogue and 2021 China Torch Statistical Yearbook(URL: http://www.chinatorch.gov.cn/). Urban socio-economic data are derived from China City Statistical Yearbook (2021), China Environmental Statistical Yearbook (2021), China Urban Construction Statistical Yearbook (2020) and Provincial and prefectural city Statistical Yearbook (2021) (URL: http://www.stats.gov.cn/). Distance data are derived from the mileage required for driving between HTZs retrieved from Baidu Map with the restriction of “shortest distance”(URL: https://map.baidu.com/).

## Code availability

Codes for data analysis are available upon request.

## References

[1] Taylor PJ, Hoyler M, Verbruggen R (2010). External urban relational process: Introducing central flow theory to complement central place theory. Urban Stu..

[2] MANUEL C.The Rise of the Network SocietyM. *Wiley‐Blackwell* (2009).

[3] Freeman C (1991). Networks of innovators: A synthesis of research issues. Res. Policy.

[4] Kroll H, Liefner I (2007). Spin-off enterprises as a means of technology commercialisation in a transforming economy–evidence from three universities in China. Technovation.

[5] Benko G (2000). Technopoles, high-tech industries and regional development: A critical review. GeoJournal.

[6] Lin JG, Xia LL, Cai RL (2021). The knowledge bases of China's high-tech industrial development zones and their effects on innovation: A study on the listed enterprises. Geogr. Res..

[7] Zhao JJ, Li FH (2013). Evolutionary mechanism exploration and developing path design of China national high-tech innovative parks(HITP) in ecological perspective. Human Geogr..

[8] Duan DZ, Du DB, Gui QH (2018). The geography of Chinese entrepreneurial development. Human Geogr..

[9] Ma HT, Zhang FF, Liu Y (2018). Transnational elites enhance the connectivity of Chinese cities in the world city network. Environ. Plan. A: Econ. Space.

[10] Shi WT, Du DB, Yang WL (2019). The flow network of chinese scientists and its driving mechanisms based on the spatial development path of CAS and CAE academicians. Sustainability.

[11] Wang J, Du GJ (2021). Spatial association network of green innovation in Chinese cities and its impact effect. China population. Res. Environ..

[12] Liu CL, Niu CC (2019). Spatial evolution and factors of interurban technology transfer network in Northeast China from national to local perspectives. Acta Geographica Sinica.

[13] Li YC (2019). A preliminary analysis on urban innovation network of metropolitian region and its characteristics. City Plann. Rev..

[14] Huang XD, Ma HT, Miao CH (2021). Connectivity characteristics for city networks in China based on innovative enterprises. Acta Geographica Sinica.

[15] Cooke P (2002). Regional innovation systems: General findings and some new evidence from biotechnology clusters. J. Technol. Transf..

[16] Thrift N, Dicken P (1992). The organization of production and the production of organization: Why business enterprises matter in the study of geographical industrialization. Transact. Inst. Br. Geogr..

[17] Bathelt H (2004). Clusters and knowledge: Local buzz, global pipelines and the process of knowledge creation. Prog. Human Geogr..

[18] Kirat T, Lung Y (1999). Innovation and proximity. Eur. Urban Reg. Stu..

[19] Freel SM (2003). Sectoral patterns of small firm innovation, networking and proximity. Res. Policy.

[20] Huggins R, Prokop D (2017). Network structure and regional innovation: A study of university–industry ties. Urban Stu..

[21] Jiamin L, Yongheng F, Yihan C (2022). Evolution of innovation networks in industrial clusters and multidimensional proximity: A case of Chinese cultural clusters. Heliyon.

[22] Duan DZ, Du DB, Chen Y (2018). Spatial -temporal complexity and growth mechanism of city innovation network in China. Scientia Geographica Sinica.

[23] Lechner C, Dowling M (1999). The evolution of industrial districts and regional networks: The case of the biotechnology region Munich/MartinsriedJ. J. Manage. Gov..

[24] Losurdo F, Marra A, Cassetta E (2019). Emerging specializations, competences and firms' proximity in digital industries: The case of London. Papers Reg. Sci..

[25] Shao XY, Zhu JL, Liu YQ (2021). Spatial-temporal evolution and influencing factors of industrial technological innovation spatial correlation: based on data of industrial enterprises above scale. Areal Res. Dev..

[26] Harbi S, Amamou M, Anderson AR (2008). Establishing high-tech industry: The Tunisian ICT experienceJ. Technovation.

[27] Hobbs GK, Link NA, Scott TJ (2017). The growth of US science and technology parks: Does proximity to a university matter?. Ann. Reg. Sci..

[28] Renaud L, Martin R, Ingo S (2019). From networks to optimal higher-order models of complex systems. Nat. Phys..

[29] Wang XH, Yang YQ, Luo XY (2022). The spatial correlation network and formation mechanism of China's high-quality economic development. Acta Geographica Sinica.

[30] Ren HM, Ye MQ, Yu YJ (2021). Spatial structure and evolution characteristics of financial network in three major urban agglomerations of China: A case study of Beijing-Tianjin-Hebei, Yangtze river Delta and Pearl river delta. Econ. Geogr..

[31] Bathelt H (2007). Buzz-and-pipeline dynamics: Towards a knowledge-based multiplier model of clusters. Geogr. Compass.

[32] Cowan R, Jonard N, Zimmermann J-B (2007). Bilateral collaboration and the emergence of innovation networks. Manag. Sci..

[33] Shaw TA, Gilly J (2000). On the analytical dimension of proximity dynamics. Reg. Stu..

[34] Boschma R (2005). Proximity and innovation: A critical assessment. Reg. Stud..

[35] Carrincazeaux C, Lung Y, Rallet A (2001). Proximity and localisation of corporate R&D activities. Res. Policy.

[36] Carrincazeaux C, Lung Y, Vicente J (2008). The scientific trajectory of the French school of proximity: Interaction- and institution-based approaches to regional innovation systems. Eur. Plan. Stud..

[37] Qin Q, Wu L, Li F (2018). A social- network- based study on geo-relations in Southeast Asia. Acta Geogr. Sinica.

